# Relationship between vitamin B6 intake and thyroid function in US adults: NHANES 2007–2012 results

**DOI:** 10.1371/journal.pone.0321688

**Published:** 2025-04-16

**Authors:** Lei Li, Jiangbo Wang, Jianping Chen

**Affiliations:** Department of Thyroid and Breast Surgery, Yijishan Hospital, First Affiliated Hospital of Wannan Medical College, Wuhu, Anhui, P.R. China; King Abdulaziz University Faculty of Medicine, SAUDI ARABIA

## Abstract

**Background:**

Existing studies have focused on the relationship between vitamin B6 and thyroid disease. However, there is a lack of large cross-sectional studies reporting on the relationship between vitamin B6 and thyroid function. Therefore, the present study aimed to assess the association between vitamin B6 intake and thyroid function in a population of US adults aged 20 years and older, using data from the National Health and Nutrition Examination Survey (NHANES) between 2007 and 2012.

**Methods:**

Demographic, dietary, thyroid function, and relevant data from NHANES 2007–2012 were collected. The relationship between vitamin B6 intake and thyroid function was analysed using weighted multiple regression and restricted cubic spline analysis, including subgroup and interaction analysis.

**Results:**

The study included 6954 participants with a weighted mean age of 47.39  ± 16.60 years, mean vitamin B6 intake of 2.07 ± 1.15 mg, and mean TT4 level of 7.88 ± 1.61 μg/dL. A statistically significant negative correlation was noted between vitamin B6 intake and TT4 levels (β  =  −0.05, 95% CI  =  −0.10 to 0.00, P  =  0.033). In addition to this, subgroup analyses we found: In the gender subgroup, a significant negative correlation was found between vitamin B6 intake and TT4 levels in the male population (β = −0.06, 95% CI  =  −0.11 to −0.01, P  =  0.028); in the age subgroup, a significant negative correlation was found between vitamin B6 intake and TT4 levels in older people aged 60–80 years (β  =  −0. 13, 95% CI  =  −0.23 to −0.04, P  =  0.008); in the BMI subgroup, we found a significant negative correlation between vitamin B6 intake and TT4 levels in overweight people (BMI: 25–29.9 kg/m^2^) (β  =  −0.13, 95% CI  =  −0.20 to −0.06, P < 0. 001); in the iodine content subgroup, we found a significant negative correlation between vitamin B6 intake and TT4 levels in people with a normal iodine intake (100–299 ug/L) (β  =  −0.07, 95% CI  =  −0.13 to −0.01, P  =  0.021). Finally, in the subgroups of gender, age, BMI, and iodine content, no interaction was found.

**Conclusions:**

To summarise, we believe that vitamin B6 may reduce serum TT4 levels by inhibiting inflammation.

## Introduction

Vitamin B6, also known as pyridoxine, is a water-soluble nutrient crucial for human health and well-being [1]. Its active form, pyridoxal 5’-phosphate (PLP), serves as a cofactor in >150 enzymatic reactions and plays a role in inflammatory processes [2–4]. Studies have indicated a negative correlation between vitamin B6 intake and various inflammation markers [5]. Furthermore, both vitamin B6 intake and supplementation have been associated with improved immune function, particularly in individuals with vitamin B6 deficiency [6,7].

The thyroid gland is a pivotal endocrine organ governing human growth, development, and metabolism through the secretion of thyroid hormones (THs) [8–10]. This process is intricately regulated by the hypothalamus–pituitary–thyroid axis, where thyroid stimulating hormone (TSH) synthesised by the pituitary gland directly influences TH synthesis and secretion. TSH serves as a crucial indicator for subclinical hyperthyroidism and hypothyroidism. TH synthesis commences with iodine oxidation, leading to the formation of thyroglobulin derivatives (MIT and DIT) and culminating in the production of T3 and T4 via parallel coupling [11]. The enzyme thyroid peroxidase (TPO) plays a central role in this synthesis process. Free TH (FT3 and FT4) constitutes the biologically active forms of TH, with total TH (TT3 and TT4) converted simultaneously into free forms to maintain physiological levels [8].

While iodine intake significantly impacts thyroid function [12], dietary nutrients also play a crucial role [13]. Selenium intake, for instance, has been linked to reduced risk of Hashimoto’s thyroiditis, an immune system-induced chronic inflammatory disease of the thyroid gland characterised by chronic lymphocytic infiltration and abnormally high levels of TGAb and TPOAb antibodies [14]. Thyroid function is also influenced by zinc, iron, and vitamin D intake [15–17]. The relationship between vitamin B6 and thyroid disease has been the focus of recent research. Researchers found that TSH secretion via the hypothalamus/pituitary gland was suppressed by intravenous vitamin B6 in patients with primary hypothyroidism in a double-blind, randomised, controlled clinical trial [18]. In addition, a controlled study showed that patients with Hashimoto’s thyroiditis had slightly lower serum vitamin B6 levels (but no statistically significant difference) and higher levels of free thyroid hormone than healthy controls. This suggests that monitoring vitamin B6 levels may help to control Hashimoto’s thyroiditis [19]. However, the specific relationship between vitamin B6 and thyroid function has not been investigated in large cross-sectional studies. In an animal study, researchers found that serum T3 and T4 and pituitary TSH levels were significantly reduced in rats in the vitamin B6-deficient group compared with rats in the normal and vitamin B6-supplemented groups, but there was no significant difference in serum TSH [20]. Therefore, our study aims to investigate this association among individuals aged ≥20 years using data from the National Health and Nutrition Examination Survey (NHANES) spanning from 2007 to 2012.

## Methods

### Population and design of the study

**Fig 1:**
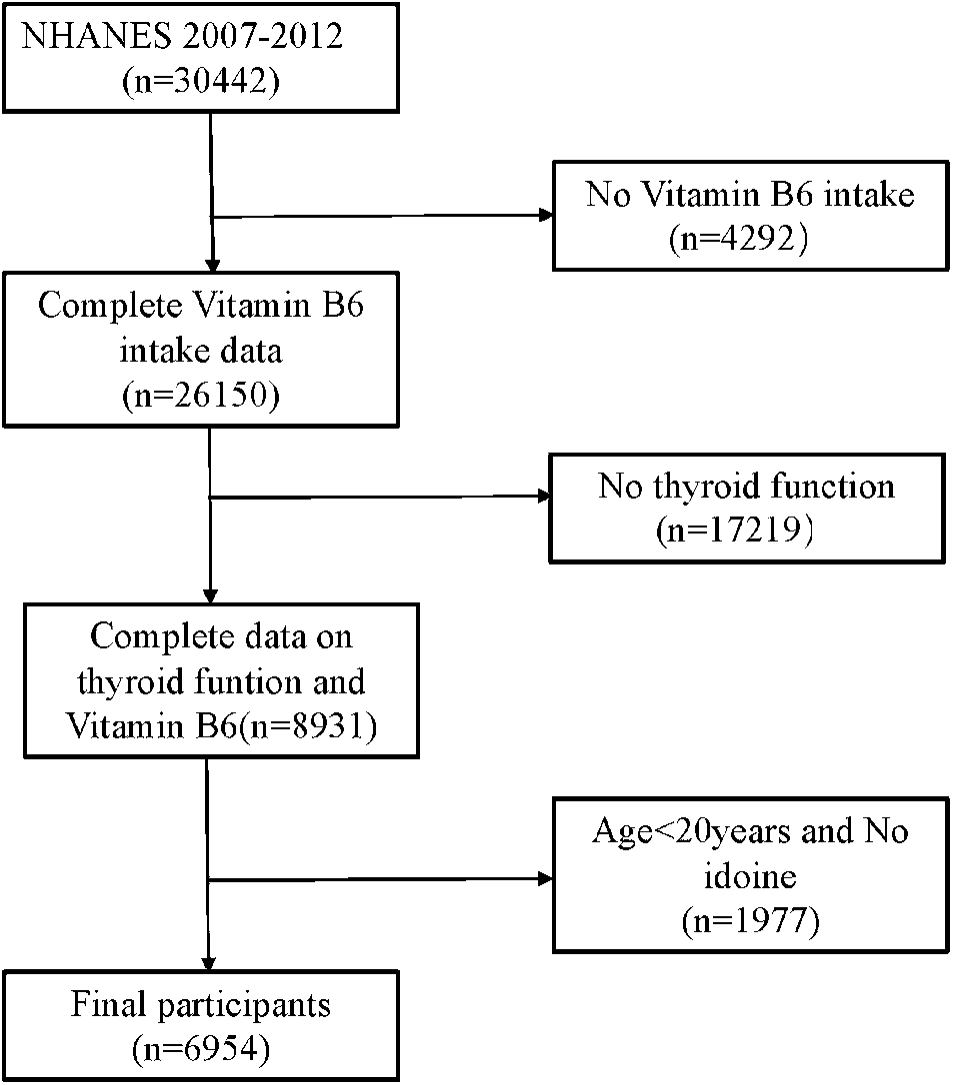
Screening process for NHANES participants from 2007 to 2012

NHANES is a research programme conducted by the National Center for Health Statistics (NCHS) to evaluate the health and nutritional status of adults and children in the United States. Data collection involved interviews at participants’ homes and health measurements in specialised mobile centres to ensure the reliability of data collection. Our study utilised three NHANES cross-sections from 2007–2008, 2009–2010, and 2011–2012, approved by the NCHS Research Ethics Review Board (ERB). In this study, we selected dietary vitamin B6 data and thyroid function laboratory data. We excluded data from individuals with missing iodine data and age <20 years. A total of 7936 participants were included in our analyses (Fig 1).

### Evaluation of dietary vitamin B6

Dietary vitamin B6 served as the primary predictor variable. For the analysis, we used data from two sets of dietary recall interviews. The first 24 h dietary recall data were collected at the Mobile Examination Centre (MEC), and the second 24 h dietary recall data were obtained through a follow-up interview conducted by telephone 3 to 10 days later. The average of the two 24 h dietary recalls was used as the final data for vitamin B6. The method of the 24 h dietary recall was based on the NHANES protocol as described in detail in the Dietary Intake Survey Procedures Manual [21–23].

### Thyroid gland function

Thyroid function, our outcome variable, encompassed eight measures assessed via serum immunoenzymatic assay: TSH, T3/FT3, T4/FT4, TT3(Total Triiodothyronine), TT4(Total Thyroxine), thyroglobulin, thyroglobulin antibody, and thyroid peroxidase antibody. Sample collection and processing adhered to NHANES Laboratory/Medical Technician Procedure Manual guidelines.

### Covariates

Covariates included age, gender, race, education, annual household income and poverty ratio, marital status, smoking, urinary iodine concentration (UIC), body mass index (BMI), energy intake, dietary fibre, and total vitamin B6 supplementation. Following the NHANES article on thyroid function, we classified BMI as normal (BMI < 25 kg/m^2^), overweight (25 kg/m^2^ ≤ BMI < 30 kg/m^2^), and obese (BMI ≥ 30 kg/m^2^); smoking as never, sometimes, and daily; and urinary iodine as iodine deficiency (UIC < 100μg/L), normal iodine (100 μg/L ≤ UIC < 300μg/L), and iodine excess (UIC ≥ 300μg/L) [13,24]. Table 1 presents detailed breakdowns.

**Table 1:** Baseline characteristics of participants in 2007–2012 (weighted).

		Vitamin B6				
Characteristic	Overall	Q1(<1.27mg)	Q2(1.27–1.76mg)	Q3 (1.76–2.43mg)	Q4(>2.43mg)	p-value
	(n = 6954)	(n = 1741)	(n = 1736)	(n = 1738)	(n = 1739)	
**Gender(%)**						<0.001
Female	3553 (53.3%)	1197 (74.2%)	1024 (62.1%)	807 (50.5%)	525 (31.3%)	
male	3401 (46.7%)	544 (25.8%)	712 (37.9%)	931 (49.5%)	1214 (68.7%)	
**Mean ± SD**	47.39 ± 16.60	47.91 ± 17.39	48.62 ± 16.81	48.26 ± 16.18	45.07 ± 15.92	<0.001
**Age(yrs)**						
**Race(%)**						0.03
Mexican American	1084 (7.8%)	269 (7.8%)	266 (7.4%)	274 (8.3%)	275 (7.5%)	
Other Hispanic	731 (5.1%)	206 (5.7%)	200 (5.4%)	173 (5.0%)	152 (4.4%)	
Non-Hispanic White	3332 (71.2%)	777 (68.2%)	818 (71.1%)	827 (70.4%)	910 (74.4%)	
Non-Hispanic Black	1378 (10.2%)	396 (12.9%)	359 (10.7%)	328 (9.4%)	295 (8.3%)	
Other	429 (5.7%)	93 (5.4%)	93 (5.4%)	136 (6.8%)	107 (5.4%)	
**Education level (%)**						<0.001
Elementary and Middle School	784 (5.7%)	292 (9.2%)	193 (5.7%)	166 (4.9%)	133 (3.7%)	
High School	1118 (12.3%)	350 (16.3%)	282 (11.8%)	238 (10.8%)	248 (10.8%)	
High School Graduate	1627 (23.9%)	414 (25.0%)	422 (26.0%)	415 (23.6%)	376 (21.3%)	
College	1932 (30.3%)	443 (30.4%)	475 (29.0%)	524 (33.2%)	490 (28.8%)	
College Graduate	1490 (27.7%)	241 (19.0%)	363 (27.3%)	394 (27.4%)	492 (35.4%)	
Missing	3 (0.1%)	1 (0.0%)	1 (0.2%)	1 (0.0%)	0 (0.0%)	
**Mean ± SD**	2.99 ± 1.66	2.57 ± 1.66	2.96 ± 1.62	3.15 ± 1.63	3.21 ± 1.66	<0.001
**Ratio of family income to poverty**						
**Marital status (%)**						0.003
Married	3726 (56.4%)	854 (51.3%)	907 (55.1%)	983 (59.9%)	982 (58.3%)	
Widowed	592 (6.0%)	198 (8.6%)	162 (6.9%)	144 (5.9%)	88 (3.1%)	
Divorced	736 (10.0%)	224 (11.4%)	191 (10.4%)	164 (9.5%)	157 (8.8%)	
Separated	225 (2.3%)	73 (3.3%)	51 (2.0%)	49 (1.9%)	52 (2.0%)	
Never married	1159 (18.0%)	280 (17.7%)	296 (18.1%)	262 (15.2%)	321 (20.7%)	
Living with partner	515 (7.4%)	112 (7.7%)	128 (7.4%)	136 (7.5%)	139 (7.0%)	
Missing	1 (0.0%)	0 (0.0%)	1 (0.0%)	0 (0.0%)	0 (0.0%)	
**Mean ± SD**	28.69 ± 6.58	28.80 ± 6.67	28.91 ± 7.00	28.92 ± 6.41	28.19 ± 6.24	0.03
**BMI**						
**Mean ± SD**	98.35 ± 16.01	97.54 ± 15.85	98.23 ± 16.76	99.27 ± 15.29	98.25 ± 16.08	0.15
**Waist circumference**						
**Mean ± SD**	5.12 ± 1.07	5.13 ± 1.09	5.13 ± 1.07	5.12 ± 1.06	5.11 ± 1.07	0.6
**Total cholesterol (mmol/L)**						
**Smoking status(%)**						<0.001
Never	1814 (25.1%)	410 (21.8%)	452 (25.3%)	470 (25.0%)	482 (27.7%)	
Fomer smoking	234 (3.8%)	54 (4.4%)	50 (2.8%)	64 (3.3%)	66 (4.9%)	
Current smoking	1179 (17.4%)	380 (24.9%)	288 (17.6%)	246 (14.5%)	265 (13.9%)	
Missing	3727 (53.7%)	897 (48.9%)	946 (54.2%)	958 (57.3%)	926 (53.6%)	
**Mean ± SD**	3.16 ± 0.42	3.13 ± 0.40	3.14 ± 0.53	3.15 ± 0.36	3.22 ± 0.38	<0.001
**T3 (pg/mL)**						
**Mean ± SD**	10.26 ± 2.01	10.39 ± 2.08	10.33 ± 2.05	10.20 ± 1.95	10.15 ± 1.97	0.02
**T4 (pmol/L)**						
**quartile**						
**TSH (mIU/L)**	1.63 [1.08, 2.42]	1.58 [1.02, 2.34]	1.60 [1.06, 2.45]	1.62 [1.09, 2.47]	1.67 [1.13, 2.45]	0.13
**quartile**						
**Thyroid peroxidase antibodies (IU/mL)**						
	0.60 [0.30, 1.70]	0.60 [0.30, 1.40]	0.60 [0.30, 1.90]	0.70 [0.36, 1.70]	0.70 [0.40, 1.80]	0.79
**Mean ± SD**						
**Total T3 (ng/dL)**	1.79 ± 0.47	1.82 ± 0.51	1.77 ± 0.46	1.77 ± 0.44	1.80 ± 0.45	0.44
**Mean ± SD**	7.88 ± 1.61	8.24 ± 1.74	7.94 ± 1.57	7.82 ± 1.56	7.58 ± 1.52	<0.001
**Total T4 (μg/dL)**						
**Mean ± SD**	2133.30 ± 989.24	1488.55 ± 636.72	1864.66 ± 703.72	2213.17 ± 798.67	2816.77 ± 1147.36	<0.001
**Energy(Kcal)**						
**Mean ± SD**	16.74 ± 10.28	10.55 ± 5.95	14.34 ± 7.61	17.90 ± 8.80	22.78 ± 12.54	<0.001
**Dietary fiber(g)**						
**UIC(%)**						0.9
Iodine deficient	2196 (32.9%)	553 (34.0%)	548 (31.8%)	551 (33.0%)	544 (32.9%)	
(<100 ug/L)						
Normal (100–299 ug/L)	3407 (48.3%)	841 (46.9%)	856 (49.3%)	848 (47.4%)	862 (49.3%)	
Excessive iodine intake	1351 (18.8%)	347 (19.1%)	332 (19.0%)	339 (19.6%)	333 (17.8%)	
(≥300 ug/L)						
**BMI(%)**						0.06
Normal (<25 kg/m^2^)	1960 (30.9%)	473 (31.1%)	461 (30.8%)	479 (28.6%)	547 (32.9%)	
Overweight	2367 (33.5%)	572 (31.1%)	578 (31.8%)	615 (34.9%)	602 (35.7%)	
(25–29.9 kg/m^2^)						
Obese (≥30 kg/m^2^)	2572 (34.9%)	678 (36.8%)	680 (36.6%)	634 (36.1%)	580 (30.7%)	
Missing	55 (0.7%)	18 (1.0%)	17 (0.8%)	10 (0.5%)	10 (0.7%)	
**Mean ± SD**	2.07 ± 1.15	0.96 ± 0.23	1.51 ± 0.14	2.07 ± 0.19	3.45 ± 1.25	<0.001
**Vitamin B6**						
**Mean ± SD**	2.06 ± 1.44	0.95 ± 0.40	1.48 ± 0.46	2.07 ± 0.64	3.45 ± 1.89	<0.001
**First Vitamin B6**						
**Mean ± SD**	2.08 ± 1.36	0.97 ± 0.40	1.55 ± 0.45	2.08 ± 0.63	3.44 ± 1.72	<0.001
**Second Vitamin B6**						
**agegroup**						<0.001
Age(20–39yrs)	2363 (37.1%)	527 (36.6%)	566 (34.1%)	583 (34.1%)	687 (42.8%)	
Age(40–59yrs)	2294 (39.4%)	545 (37.0%)	567 (39.9%)	580 (41.4%)	602 (39.1%)	
Age(60–80yrs)	2297 (23.5%)	669 (26.4%)	603 (25.9%)	575 (24.5%)	450 (18.0%)	
**quartile**						
**Total vitamin B6 supplement(mg)**						
	2.60 [2.00, 5.00]	2.58 [2.00, 5.00]	2.50 [1.82, 5.08]	2.60 [2.00, 4.77]	2.80 [2.00, 6.00]	0.7
**quartile**			1			
**UIC** (**μg/L**)	146.80 [82.10, 250.40]	139.48 [79.51, 248.48]	45.21 [83.52, 248.53]	145.25 [80.40, 257.13]	152.98 [83.02, 248.28]	0.54
**quartile**						
**Thyroglobulin antibodies (IU/mL)**						
	0.60 [0.60, 0.60]	0.60 [0.60, 0.60]	0.60 [0.60, 0.60]	0.60 [0.60, 0.60]	0.60 [0.60, 0.60]	0.5
**quartile**						
**Thyroglobulin (ug/L)**	10.01 [5.48, 17.43]	11.02 [5.50, 18.86]	9.68 [5.35, 18.10]	9.91 [5.46, 15.86]	9.71 [5.61, 17.02]	0.05

^1^For continuous variables: survey-weighted Mean (SD) or survey-weighted Median(IQR), P-value was by survey-weighted linear regression (svyglm). For categorical variables: N-observe (survey-weighted percentage), P-value was by survey-weighted Chi-square test (svytable).

BMI: body mass index; UIC: urinary iodine concentration, a measure of iodine status.

Vitamin B6 supplementation indicates the use of vitamin B6 supplements by the participants over a 1-month period. Continuous variables are presented as weighted mean (SD), and categorical variables as weighted percentage.

### Statistical analyses

We applied NHANES guidelines for sampling weights and employed complex multistage sampling procedures to prevent oversampling bias. Continuous variables are expressed as mean  ±  standard deviation, and categorical variables as frequencies and percentages. We utilised weighted multiple linear regression models, including unadjusted, partially adjusted, and fully adjusted models, to assess the association between vitamin B6 intake and thyroid function. The partially adjusted model was adjusted for age, gender, ethnicity, education level, and UIC. Fully adjusted models included age, gender, race, education level, marital status, annual household income and poverty ratio, UIC, smoking, energy intake, dietary fibre, and BMI. Interaction and subgroup analyses were conducted for Age, Gender, BMI and UIC. Restricted cubic spline (RCS) analysis was used to construct regression curves. Statistical analyses were performed using software packages R (http://www.R-project.org; R Foundation) and EmpowerStats (www.empowerstats.com).

### Ethics statement

The studies involving human participants received approval from the National Center for Health Statistics (NCHS) and the Research Ethics Review Board. The research was conducted in compliance with local legislation and institutional requirements. Participants provided their written informed consent to take part in this study.

## Results

### Baseline characteristics of the study population

A total of 6954 participants were included, with a mean age of 47.39  ± 16.60 years. Gender distribution was relatively balanced, comprising 53.3% Females and 46.7% males. Stratification of the results by vitamin B6 quartiles revealed significant differences in dietary (energy intake and dietary fibre; P < 0.05) and laboratory (FT3, FT4, TT4, and TG; P < 0.05) data, as well as demographic data such as smoking status, marital status, annual household income and poverty ratio, education level, age, and gender (all P < 0.05; Table 1). Notably, the highest vitamin intake group (Q4) had significantly lower TT4 levels, more females, higher education levels, increased energy and fibre intake than the lowest intake group (Q1).

### Relationship between vitamin B6 intake and thyroid function

Weighted multivariable linear regression revealed correlations between vitamin B6 intake and thyroid function (Table 2). In the unadjusted model, positive correlations were observed with T3 (β  =  0.03, 95% CI  =  0.02–0.04, P  =  0.001) and negative correlations with TT4 and TG (TT4: β  =  −0.17, 95% CI  =  −0.22 to −0.13, P < 0.001; TG: β  =  −0.99, 95% CI  =  −1.7 to −0.24, P = 0.011). Compared with model 1, model 2 reported a negative correlation only between TT4 and vitamin B6 intake (β  =  −0.10, 95% CI  =  −0.14 to −0.06, P < 0.001), after adjusting for age, gender, ethnicity, education level and UIC. In model 3, we further adjusted for marital status, annual household income and poverty ratio, smoking, energy intake, dietary fibre, and BMI and found that TT4 is negatively associated with vitamin B6 intake (TT4: β  =  −0.05, 95% CI  =  −0.10 to 0.00, P  =  0.033). In addition, we examined the correlation between vitamin B6 supplementation and thyroid function, and the results showed no correlation between vitamin B6 supplementation and these 8 indicators of thyroid function. (Supplyment, S1 Table).

### Relationship between vitamin B6 intake and thyroid function: RCS analysis

**Fig 2:**
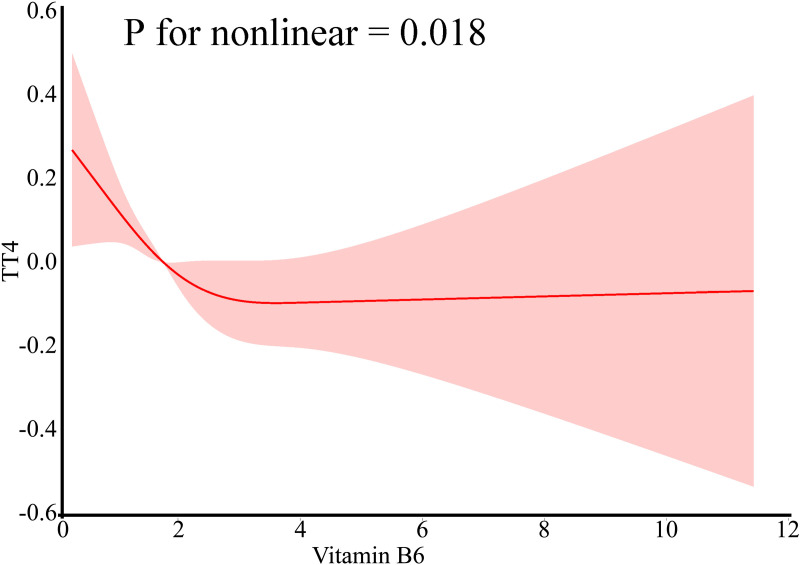
Non-linear relationship between vitamin B6 intake and eight thyroid functions based on RCS regression models: (A)Vitamin B6 intake andTT4.

RCS curves for the modelling and visualisation of the association between vitamin B6 and eight indicators of thyroid function demonstrated a non-linear relationship between vitamin B6 and TT4 levels after full adjustment for confounders in model 3 (TT4: P for non-linear  =  0.018). The results showed that among these eight indicators of thyroid function, only TT4 and vitamin B6 were found to correlate significantly (Fig 2). As shown in the appendix (Supplyment, S1 Fig), there was no correlation between the remaining 7 indicators of thyroid function and vitamin B6. We also analysed the relationship between vitamin B6 supplement intake and thyroid function by RCS. This showed no correlation between these 8 indicators of thyroid function and vitamin B6 supplement intake, as shown in the appendix (Supplyment, S2 Fig).

### Subgroup analysis

In subgroup analyses according to gender, men had a more significant negative correlation between vitamin B6 intake and TT4 levels compared to female adults (β  =  −0.06, 95% CI  =  −0.11 to −0.01, P  =  0.028); in subgroup analyses according to age, older adults (age: 60–80 years) had a more significant negative correlation between vitamin B6 intake and TT4 levels compared to the other agegroups (β  =  −0. 13, 95% CI  =  −0.23 to −0.04, P  =  0.008); in the subgroup analysis of BMI, there was a more significant negative correlation between vitamin B6 intake and TT4 levels in the overweight population (BMI: 25–29.9 kg/m2) (β  =  −0. 13, 95% CI  =  −0.20 to −0.06, P  =  0.001); in the UIC subgroup, vitamin B6 intake and TT4 levels were more negatively correlated in those with normal iodine intake (β  =  −0.07, 95% CI  =  −0.13 to −0.01, P  =  0.021) (Tables 3). The interaction analysis did not show any significant interactions between gender, age, BMI, UIC and the relationship between vitamin B6 and TT4 levels (P for interaction > 0.05; Tables 3).

## Discussion

This cross-sectional study included 6954 US adults aged ≥20 years from NHANES 2007–2012. We employed weighted multiple regression and RCS to explore the association between vitamin B6 intake and thyroid function and found a significant negative correlation between vitamin B6 intake and TT4 levels, particularly notable in the highest intake group (Q4) compared with the lowest intake group (Q1) in the fully adjusted model. RCS analyses further supported a non-linear negative correlation between vitamin B6 intake and TT4 levels. Gender, Age, BMI and UIC are known to influence thyroid function significantly. Thus, we conducted subgroup analyses stratified by Gender, Age, BMI and UIC to assess the relationship between vitamin B6 intake and TT4. Notably, the negative correlation between vitamin B6 intake and TT4 levels was more pronounced in male, overweight, older age groups (60–80 years), and individuals with normal iodine intake. An interaction analysis between vitamin B6 intake and gender, age, BMI and iodine levels demonstrated no correlation between vitamin B6 intake and TT4 levels in these subgroups (P > 0.05).

To the best of our knowledge, this is the first study to investigate the relationship between dietary B6 intake and thyroid function.Both iodine and tyrosine are raw materials for TH synthesis; thus, iodine intake certainly affects thyroid function. However, the impact of dietary factors other than iodine has also been highlighted in epidemiological studies. For instance, increased dietary selenium intake was negatively correlated with TT4 and TT4/TT3 in a study among US adults, and selenium is known for its anti-inflammatory properties that maintain TH levels and support thyroid function [25]. Moreover, a study in US adult males reported significant associations between the Dietary Inflammatory Index (DII) and increased TT4 and FT3 levels ([13]). The DII Disorder Number assesses the body’s inflammatory potential through dietary intake and predicts higher IL-6 and C-reaction protein(CRP) levels [26,27]. In large cross-sectional studies evaluating the immune-inflammatory index and thyroid function, the SII (immune-inflammatory index) was reported to be significantly negatively correlated with FT3 and FT3/FT4 and significantly positively correlated with TT4 [28]. These studies are mutually supportive of the important role of inflammation in the regulation of thyroid function.Although the mechanisms underlying the negative association between vitamin B6 intake and thyroid function remain unclear, our finding of a negative association between vitamin B6 intake and TT4 levels is biologically plausible. Tyrosine metabolism is involved in TH synthesis. Vitamin B6 is a cofactor that is directly or indirectly involved in TH synthesis [29]. Furthermore, higher vitamin B6 intake has been linked with reduced inflammation, as evidenced by its negative correlation with CRP in a large cross-sectional study in the United States and in a cross-sectional study in Boston, which reported that low serum vitamin B6 levels were associated with inflammation, higher oxidative stress, and metabolic status in Puerto Rican older adults [30,31]. The kynurenine (tryptophan-kynurenine) pathway (KP) is the main pathway for tryptophan metabolism, in which vitamin B6 plays a key role [32,33]. KP plays an important role in various immune and inflammatory mechanisms, particularly kynurenine (KYNA), which has anti-inflammatory, pro-inflammatory, and immunosuppressive properties [34–36]. In a study of female patients with autoimmune thyroiditis, a negative correlation was found between serum KYNA levels and serum deiodinase levels. Deiodinase is involved in the biological process of converting free T4 to free T3 in the serum [37]. Therefore, we suggest that vitamin B6 may be able to downregulate serum TT4 levels through a mechanism that inhibits inflammation onset. In addition, the results of the present study showed a negative correlation between the intake of vitamin B6 and the level of TT4. Thyroid hormone deficiency and excess can lead to adverse outcomes. These include hypothyroidism, hyperthyroidism and thyroid nodules [38,39]. A previous controlled trial has shown that intravenous vitamin B6 is effective in the suppression of TSH secretion in patients with hypothyroidism [39]. In addition, in the process of inflammation, vitamin B6 plays an important role. Therefore, in a population with thyroid disease and excess thyroid hormone, we hypothesised that vitamin B6 supplementation would probably be beneficial. However, to confirm the efficacy and safety of vitamin B6 in treating patients with thyroid disease, further clinical trials are needed.

T3 showed a positive correlation with vitamin B6 in the unadjusted model. This correlation disappeared in the adjusted model. The possible reasons for this are as follows: 1. Uncontrolled confounding may have caused the positive association observed in the unadjusted model. For example, both vitamin B6 intake and T3 levels may be influenced by factors such as lifestyle, intake of other nutrients and health status. When these variables are included in the adjusted model, the original correlation may become weaker or disappear.2. Initial positive correlations may be found by chance (i.e., false positives) and are particularly likely to occur in initial analyses involving large numbers of unadjusted variables. The inclusion of additional control variables may reveal that the initial correlation is not statistically significant as model adjustments become deeper.

We found a negative association between dietary vitamin B6 intake and TT4 levels in male adults in a gender subgroup analysis. Previous studies have shown that oestrogen increases iodine uptake and has an effect on the redox state of the thyroid [40–42]. In contrast, the effect of vitamin B6 on thyroid hormones is less pronounced and would therefore be more important in men. However, this requires further research and investigation. It has previously been shown that thyroid hormone secretion declines with age, possibly due to age-related changes in the enzymes involved in TH synthesis [43], and in age subgroup analyses we found a negative association between vitamin B6 intake and TT4 levels in the elderly population. We found a negative association between vitamin B6 intake and TT4 levels in overweight individuals in BMI subgroup analyses. In a previous cross-sectional study of mildly overweight people with normal thyroid function, an association was found between accumulation of abdominal subcutaneous fat and decreased T4 and TSH levels [44]. This is similar to our findings. However, the association between BMI and vitamin B6 and thyroid hormones needs further investigation. We found a negative correlation between vitamin B6 and TT4 levels in the iodine-normal group in a subgroup analysis of UIC. Previous studies have shown that both iodine excess and deficiency in humans can affect thyroid function. Thus, abnormal iodine levels in humans may reduce the effect of vitamin B6 on thyroid function [45,46].

In addition to this, the results of our study showed that there was no significant correlation between the intake of food supplements and the eight tests related to thyroid function. Possible reasons for this are:1. Vitamin B6 in supplements may differ from vitamin B6 in food in terms of bioavailability and absorption, which may affect its potential effect on thyroid function.2. The doses of supplements used in the study may not have been sufficient to have an effect on thyroid function or may have been so high that they could not be effectively utilised in vivo.3. If the participants already had an adequate intake of vitamin B6, additional supplements may not have produced any additional physiological effects.4. It is possible that the sample size was not large enough, resulting in a study with insufficient statistical power to detect small effect sizes. Discuss this point and recommend an increase in sample size in future trials.5. Consider a long-term follow-up study to assess the effects of long-term vitamin B6 supplementation on thyroid function.

Despite the strengths of our study, such as a nationally representative sample in the United States, which increases the generalisability of our results, and careful adjustment for confounding variables, several limitations should be noted. The cross-sectional nature of the study meant that it was not possible to establish any cause-and-effect relationship between vitamin B6 intake and thyroid function, and future studies are needed to clarify the potential mechanism of vitamin B6 and thyroid function. Second, although the present study estimated vitamin B6 intake by averaging data from two dietary recalls, a method that reduces the random error of one-time measurements, large variations between individuals may still affect the accuracy of the data and may not necessarily reflect an individual’s regular intake. However, with a large sample size, these random errors can be cancelled out to some extent, resulting in more reliable statistical results. In addition, it is important to note that the biases inherent in self-reported dietary data, such as the tendency of respondents to report intakes that are lower than their actual intakes, are difficult to avoid completely [47]. Lastly, our study focused solely on US adults aged ≥20 years, excluding children.

## Conclusion

Although the present study has shown an association between vitamin B6 and serum TT4 levels, we have not been able to infer a causal relationship from this observational study. This hypothesis should be the subject of future studies through the design of intervention trials. The dose-response relationship of vitamin B6 should be further investigated on this basis. In addition, this study did not include people under the age of 20. We suggest that future studies should be expanded to include this age group. This will not only improve the generalisability of the study. It will also help to understand the relationship between vitamin B6 and thyroid function at different stages of growth and development.

**Table 2:** Relationship between vitamin B6 intake and thyroid function.

		Model 1(β (95% CI))	P Value	Model 2(β (95% CI))	P Value	Model 3(β (95% CI))	P Value
TGAb	Continuous	−0.61（−2.2, 1.0）	0.5	0.07（−1.6, 1.7）	>0.9	0.31（−1.3, 2.0）	0.7
	Q1	Ref		Ref		Ref	
	Q2	9.2（0.69, 18）	0.035	9.1（0.77, 17）	0.033	9.3（0.61, 18）	0.037
	Q3	0.70（−2.5, 3.9）	0.7	0.75（−2.8, 4.3）	0.7	1.4（−3.0, 5.8）	0.5
	Q4	1.6（−3.3, 6.5）	0.5	3.4（−1.5, 8.3）	0.2	4.4（−1.3, 10）	0.12
T3	Continuous	0.03(0.02, 0.04)	<0.001	0.00(−0.01, 0.01)	0.6	0.00(−0.01, 0.01)	>0.9
	Q1	Ref		Ref		Ref	
	Q2	0.00(−0.03, 0.03)	0.8	−0.01(−0.04, 0.02)0.6		−0.01(−0.04, 0.03)	0.7
	Q3	0.02(−0.02, 0.05)	0.3	−0.02(−0.05, 0.02)	0.3	−0.02(−0.05, 0.02)	0.4
	Q4	0.09(0.05, 0.12)	<0.001	−0.01(−0.05, 0.02)0.5		−0.01(−0.04, 0.03)	0.8
T4	Continuous	−0.05(−0.12, 0.02)	0.14	−0.04(−0.11, 0.04)0.3		0.00(−0.08, 0.07)	>0.9
	Q1	Ref		Ref		Ref	
	Q2	−0.06(−0.26, 0.13)	0.5	−0.09(−0.29, 0.11)	0.4	−0.06(−0.27, 0.14)	0.5
	Q3	−0.19(−0.44, 0.06)	0.13	−0.23(−0.49, 0.02)	0.07	−0.16(−0.44, 0.11)	0.2
	Q4	−0.24(−0.46, −0.02)	0.036	−0.25(−0.48, −0.02)0.035		−0.16(−0.41, 0.10)	0.2
TG	Continuous	−0.99(−1.7, −0.24)	0.011	0.02(−0.55, 0.58)	>0.9	0.40(−0.24, 1.0)	0.2
	Q1	Ref		Ref		Ref	
	Q2	0.57(−5.7, 6.8)	0.9	1.6(−5.0, 8.1)	0.6	2.0(−4.4, 8.3)	0.5
	Q3	−0.44(−4.0, 3.2)	0.8	1.3(−2.4, 5.0)	0.5	2.1(−1.1, 5.3)	0.2
	Q4	−3.2(−6.0, −0.38)	0.027	0.13(−3.0, 3.3)	>0.9	1.3(−1.0, 3.6)	0.3
TSH	Continuous	−0.04(−0.11, 0.03)	0.2	−0.02(−0.08, 0.04)	0.4	−0.03(−0.09, 0.04)	0.4
	Q1	Ref		Ref		Ref	
	Q2	0.24(−0.24, 0.72)	0.3	0.22(−0.25, 0.68)	0.3	0.21(−0.27, 0.68)	0.4
	Q3	−0.03(−0.23, 0.17)	0.8	−0.04(−0.24, 0.17)0.7		−0.05(−0.25, 0.16)	0.6
	Q4	−0.08(−0.31, 0.16)	0.5	−0.05(−0.26, 0.17)	0.7	−0.07(−0.30, 0.16)	0.5
TPOAb	Continuous	0.39（−2.9, 3.7）	0.8	2.3（−1.3, 5.8）	0.2	4.3（0.29, 8.4）	0.037
	Q1	Ref		Ref		Ref	
	Q2	−1.2（−12, 9.4）	0.8	−0.49（−11, 10）	>0.9	0.93（−9.2, 11）	0.8
	Q3	−2.6（−15, 10）	0.7	−0.13（−13, 13）	>0.9	3.0（−9.6, 16）	0.6
	Q4	−1.4（−14, 11）	0.8	4.2（−9.8, 18）	0.5	10（−3.2, 24）	0.13
TT3	Continuous	0.00(−0.01, 0.01)	0.6	−0.01(−0.02, 0.00)	0.09	−0.01(−0.02, 0.00)	0.11
	Q1	Ref		Ref		Ref	
	Q2	−0.06(−0.10, −0.01)	0.016	−0.05(−0.09, 0.00)0.044		−0.05(−0.10, 0.00)	0.042
	Q3	−0.05(−0.11, 0.00)	0.061	−0.05(−0.11, 0.00)0.068		−0.06(−0.12, 0.00)	0.052
	Q4	−0.02(−0.07, 0.02)	0.3	−0.05(−0.09, 0.00)0.041		−0.06(−0.12, 0.00)	0.054
TT4	Continuous	−0.17(−0.22, −0.13)	<0.001	−0.10(−0.14, −0.06)<0.001	<0.001	−0.05(−0.10, 0.00)	0.033
	Q1	Ref		Ref		Ref	
	Q2	−0.30(−0.43, −0.17)	<0.001	−0.22(−0.35, −0.10)<0.001	<0.001	−0.19(−0.31, −0.06)	0.005
	Q3	−0.42(−0.57, −0.26)	<0.001	−0.30(−0.45, −0.14)	<0.001	−0.22(−0.38, −0.06)	0.009
	Q4	−0.66(−0.79, −0.53)	<0.001	−0.43(−0.57, −0.29)	<0.001	−0.29(−0.45, −0.13)	0.001

CI, confidence interval; B6, vitamin B6 intake; T3, free triiodothyronine; T4, free thyroid hormone; TT3, total T3; TT4, total T4; TSH, thyroid stimulating hormone; TG, thyroglobulin; TGAb, thyroglobulin antibody; TPOAb, thyroid peroxidase antibody. Model 1: unadjusted. Model 2: adjusted for age, gender, ethnicity, education level, and UIC. Model 3: adjusted for age, gender, ethnicity, education level, marital status, annual household income and poverty ratio, UIC, smoking, energy intake, dietary fibre, and BMI. We then converted the continuous variable of vitamin B6 intake into quartiles. The negative correlation between TT4 and vitamin B6 intake was even more pronounced in the unadjusted group (model 1), with an effect statistic of −0.30 (β  =  −0.30, 95% CI  =  −0.43 to −0.17, P < 0.001) for Q2, −0.42 (β  =  −0.42, 95% CI  =  −0.57 to −0.26, P < 0.001) for Q3, and −0.66 (β =  −0.66, 95% CI  =  −0.79 to−0.53, P < 0.001) for Q4. In model 2, only TT4 was negatively associated with vitamin B6 intake. In quartiles, TT4 decreased by 0.43 μg/dL for each unit increase in vitamin B6 intake level in the group with the highest vitamin B6 intake (Q4) compared with the group with the lowest vitamin B6 intake (Q1). In the fully adjusted model 3, Q4 had a decrease in TT4 of 0.29 μg/dL for each unit increase in vitamin B6 intake level compared with Q1. The results indicate a significant negative correlation between vitamin B6 intake and TT4 levels.

**Table 3:** Subgroup analyses.

		β (95% CI)	P Value	p for interaction
**Gender**	**Female**	−**0.04 (**−**0.14, 0.05)**	**0.3**	**0.24**
	**male**	−**0.06 (**−**0.11,** −**0.01)**	**0.028**	
**Age**	**Age(20–39)**	−**0.02 (**−**0.09, 0.05)**	**0.5**	**0.32**
	**Age(40–59)**	−**0.06 (**−**0.14, 0.02)**	**0.2**	
	**Age(60–80)**	−**0.13 (**−**0.23,** −**0.04)**	**0.008**	
**BMI**	**Normal (<25 kg/m2)**	−**0.02(**−**0.09, 0.05)**	**0.6**	**0.58**
	**Overweight**	−**0.13(**−**0.20,** −**0.06)**	**<0.001**	
	**(25–29.9 kg/m2)**			
	**Obese (**≥**30 kg/m2)**	**0.01(**−**0.10, 0.12)**	**0.8**	
**UIC**	**Iodine deficient**	−**0.06(**−**0.16, 0.03)**	**0.2**	**0.25**
	**(<100 ug/L)**			
	**Normal (100–299 ug/L)**	−**0.07(**−**0.13,** −**0.01)**	**0.021**	
	**Excessive iodine intake**	**0.03(**−**0.10, 0.16)**	**0.6**	
	(≥**300 ug/L)**			

Subgroup analyses of the association between vitamin B6 intake and TT4, stratified by gender of NHANES participants from 2007 to 2012. The results of the subgroup analyses were adjusted for age, ethnicity, education level, marital status, annual household income and poverty ratio, UIC, smoking, energy intake, dietary fibre, and BMI. (gender was not included in the adjustment).

Subgroup analyses of the association between vitamin B6 intake and TT4, stratified by age in NHANES participants from 2007 to 2012. The results of the subgroup analyses were adjusted for gender, ethnicity, education level, marital status, annual household income and poverty ratio, UIC, smoking, energy intake, dietary fibre, and BMI. (age was not included in the adjustment).

Subgroup analyses of the association between vitamin B6 intake and TT4, stratified by BMI in NHANES participants from 2007 to 2012. The results of the subgroup analyses were adjusted for age, gender, ethnicity, education level, marital status, annual household income and poverty ratio, UIC, smoking, energy intake, and dietary fibre. (BMI was not included in the adjustment).

Subgroup analyses of the association between vitamin B6 intake and TT4, stratified by UIC in NHANES participants from 2007 to 2012. The results of the subgroup analyses were adjusted for age, gender, ethnicity, education level, marital status, annual household income and poverty ratio, smoking, energy intake, dietary fibre, and BMI. (UIC was not included in the adjustment).

## Supporting information

S1 Fig Correlation between the remaining 7 indicators of thyroid function and vitamin B6. (TIF)

S2 Fig Correlation between 8 indicators of thyroid function and vitamin B6 supplement intake. (TIF)

S1 Table Baseline characteristics of participants based on total vitamin B6 supplementation, 2007–2012 (weighted). (DOCX)
