# Supplementary figures and images for: Relationship between vitamin B6 intake and thyroid function in US adults: NHANES 2007–2012 results
Source: PLoS One. 2025 Apr 16;20(4):e0321688. doi: 10.1371/journal.pone.0321688 (PMC12002500; doi:10.1371/journal.pone.0321688)

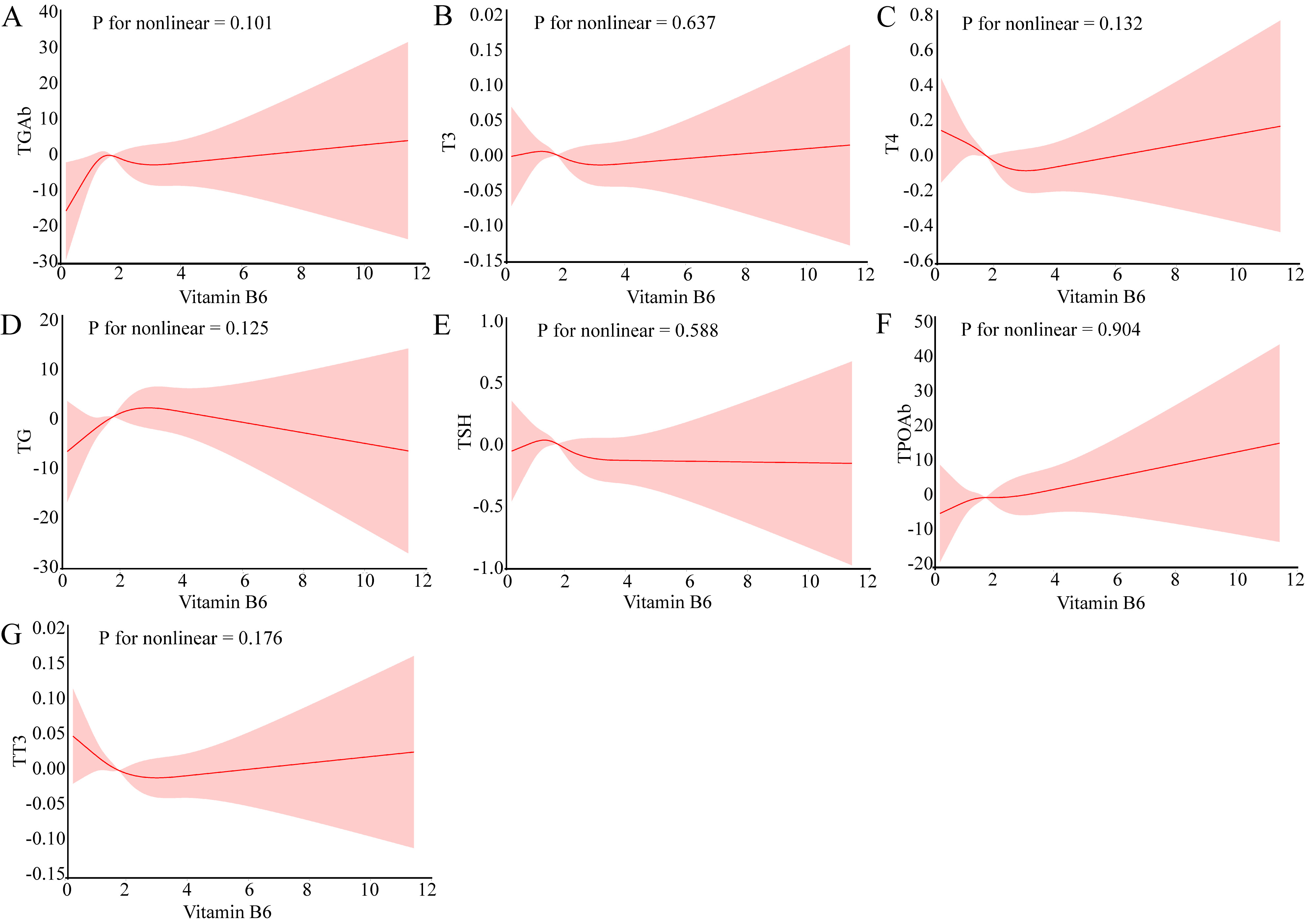

Supplement: S1 Fig — (TIF) [file pone.0321688.s001.tif]

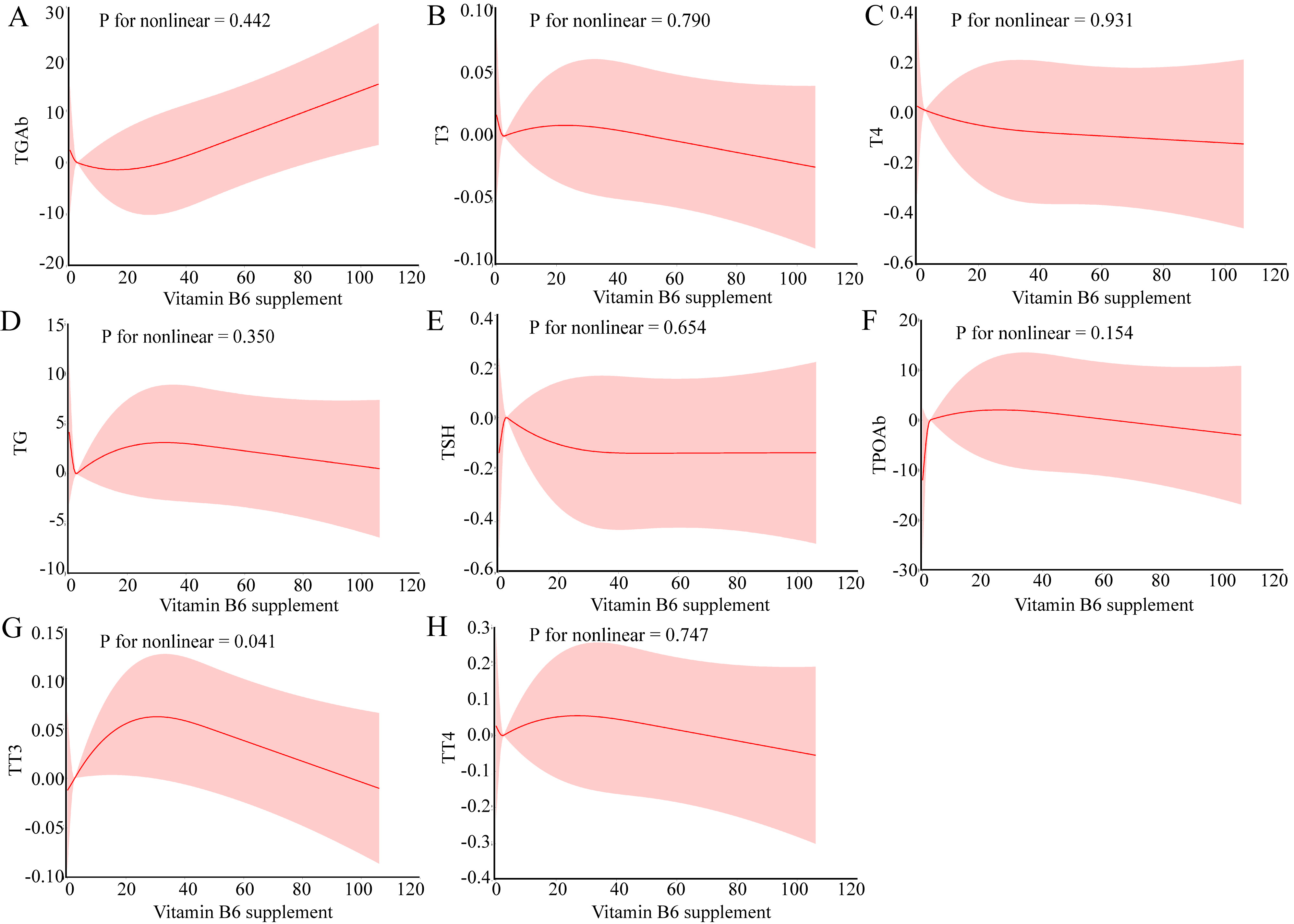

Supplement: S2 Fig — (TIF) [file pone.0321688.s002.tif]
