# Supplementary material for: Relationship between vitamin B6 intake and thyroid function in US adults: NHANES 2007–2012 results
Source: PLoS One. 2025 Apr 16;20(4):e0321688. doi: 10.1371/journal.pone.0321688 (PMC12002500; doi:10.1371/journal.pone.0321688)
Supplement: S1 Table — (DOCX) [file pone.0321688.s003.docx]

|  | | **Total vitamin B6 supplement(mg)** | | | |  |
| --- | --- | --- | --- | --- | --- | --- |
|  | **Overall (n=2448)** | **Q1(n=1133)** | **Q2(n=96）** | **Q3(n=638)** | **Q4(n=581)** | **p-value** |
| **quartile**  **Total vitamin B6 supplement(mg)** | 2.20 ±1.50 | 2.11 ±1.26 | 2.15±1.20 | 2.22±1.32 | 2.37±2.02 | 0.03 |
| **Gender(%)** |  |  |  |  |  | <0.001 |
| Female | 1324 (58.1%) | 646 (59.1%) | 73 (79.3%) | 274 (44.3%) | 331 (65.5%) |  |
| male | 1124 (41.9%) | 487 (40.9%) | 23 (20.7%) | 364 (55.7%) | 250 (34.5%) |  |
| **Mean ± SD**  **Age(yrs)** | 49.72±16.66 | 47.06±16.05 | 40.36±14.65 | 58.02±15.64 | 48.42 (16.21%) | <0.001 |
| **Race(%)** |  |  |  |  |  | 0.01 |
| Mexican American | 265 (4.4%) | 126 (4.5%) | 14 (8.0%) | 62 (4.0%) | 63 (4.0%) |  |
| Other Hispanic | 195 (3.3%) | 97 (3.8%) | 13 (6.7%) | 39 (2.2%) | 46 (3.1%) |  |
| Non-Hispanic White | 1416 (79.2%) | 630 (78.2%) | 43 (64.8%) | 395 (80.7%) | 348 (82.2%) |  |
| Non-Hispanic Black | 414 (7.5%) | 208 (8.4%) | 13 (6.9%) | 108 (7.6%) | 85 (5.8%) |  |
| Other | 158 (5.5%) | 72 (5.2%) | 13 (13.6%) | 34 (5.5%) | 39 (4.9%) |  |
| **Education level(%)** |  |  |  |  |  | 0.09 |
| Elementary and Middle School | 175 (3.3%) | 74 (2.7%) | 7 (3.9%) | 58 (5.0%) | 36 (2.6%) |  |
| High School | 246 (7.3%) | 104 (6.4%) | 8 (12.9%) | 76 (9.6%) | 58 (6.2%) |  |
| High School Graduate | 505 (19.9%) | 239 (20.3%) | 20 (15.1%) | 154 (23.5%) | 92 (16.5%) |  |
| College | 775 (32.4%) | 359 (32.3%) | 33 (35.9%) | 170 (28.9%) | 213 (35.4%) |  |
| College Graduate | 747 (37.1%) | 357 (38.3%) | 28 (32.1%) | 180 (33.1%) | 182 (39.4%) |  |
| **Mean ± SD**  **Ratio of family income to poverty** | 3.43±1.59 | 3.31±1.62 | 3.33±1.46 | 3.43±1.55 | 3.68 (1.54%) | 0.01 |
| **Marital status(%)** |  |  |  |  |  | <0.001 |
| Married | 1471 (62.8%) | 647 (60.2%) | 70 (78.6%) | 409 (67.7%) | 345 (60.4%) |  |
| Widowed | 213 (5.9%) | 88 (5.1%) | 2 (1.4%) | 82 (10.1%) | 41 (4.4%) |  |
| Divorced | 249 (9.0%) | 108 (8.4%) | 8 (8.0%) | 58 (8.4%) | 75 (10.8%) |  |
| Separated | 61 (1.8%) | 30 (2.2%) | 2 (1.2%) | 14 (1.3%) | 15 (1.8%) |  |
| Never married | 323 (14.5%) | 192 (17.9%) | 7 (4.5%) | 56 (8.3%) | 68 (15.6%) |  |
| Living with partner | 131 (6.0%) | 68 (6.3%) | 7 (6.2%) | 19 (4.4%) | 37 (7.0%) |  |
| **Mean ± SD**  **BMI** | 27.92±6.28 | 27.66±5.99 | 26.69±5.12 | 28.42±5.94 | 28.14±7.20 | 0.17 |
| **Mean ± SD**  **Waist circumference** | 96.67±15.86 | 95.69±15.22 | 93.70±14.69 | 99.90±15.47 | 95.97±17.14 | 0.17 |
| **Mean ± SD**  **Total cholesterol (mmol/L)** | 5.11±1.07 | 5.07±1.06 | 5.05±1.02 | 5.08±1.08 | 5.22±1.09 | 0.12 |
| **Smoking status(%)** |  |  |  |  |  | 0.19 |
| Never | 767 (31.3%) | 321 (30.3%) | 19 (23.7%) | 230 (35.8%) | 197 (30.1%) |  |
| Fomer smoking | 49 (2.3%) | 25 (2.4%) | 1 (0.2%) | 12 (1.5%) | 11 (3.1%) |  |
| Current smoking | 270 (10.3%) | 144 (11.2%) | 8 (6.5%) | 64 (10.7%) | 54 (8.8%) |  |
| Missing | 1362 (56.2%) | 643 (56.1%) | 68 (69.6%) | 332 (52.0%) | 319 (58.0%) |  |
| **Mean ± SD**  **Vitamin B6** | 2.11±1.38 | 2.06±1.32 | 2.24±1.18 | 2.07±1.29 | 2.22 (1.56%) | 0.11 |
| **Mean ± SD**  **T3 (pg/mL)** | 3.10±0.38 | 3.14±0.35 | 3.06±0.37 | 3.06±0.36 | 3.07±0.46 | 0.01 |
| **Mean ± SD**  **T4 (pmol/L)** | 10.29±2.08 | 10.27±1.89 | 9.84±1.51 | 10.56±2.34 | 10.15 ±2.22 | 0.97 |
| **Mean ± SD**  **Total T3 (ng/dL)** | 1.74±0.48 | 1.76±0.46 | 1.79±0.42 | 1.67±0.47 | 1.78 ±0.53 | 0.73 |
| **Mean ± SD**  **Total T4 (μg/dL)** | 7.85±1.64 | 7.81±1.52 | 7.77±1.54 | 7.80±1.61 | 7.98 ±1.86 | 0.22 |
| **Mean ± SD**  **Energy(Kcal)** | 2126.58±936.31 | 2163.13±971.33 | 2040.85±723.30 | 2074.30±915.86 | 2122.06 ±918.99 | 0.37 |
| **Mean ± SD**  **Dietary fiber(g)** | 17.86±10.09 | 17.50±10.03 | 16.95±8.11 | 17.28±9.56 | 19.22±10.83 | 0.01 |
| **UIC(%)** |  |  |  |  |  | 0.005 |
| Iodine deficient  (<100 ug/L) | 679 (30.2%) | 336 (32.6%) | 32 (39.8%) | 139 (22.2%) | 172 (31.7%) |  |
| Normal (100–299 ug/L) | 1196 (48.2%) | 560 (46.7%) | 54 (52.2%) | 313 (52.3%) | 269 (46.5%) |  |
| Excessive iodine intake (≥300 ug/L) | 573 (21.6%) | 237 (20.7%) | 10 (8.1%) | 186 (25.5%) | 140 (21.8%) |  |
| **BMI(%)** |  |  |  |  |  | 0.14 |
| Normal (<25 kg/m2) | 774 (35.5%) | 384 (37.2%) | 34 (41.3%) | 185 (28.5%) | 171 (37.6%) |  |
| Overweight (25–29.9 kg/m2) | 875 (34.7%) | 399 (34.3%) | 33 (33.4%) | 231 (38.7%) | 212 (31.8%) |  |
| Obese (≥30 kg/m2) | 786 (29.4%) | 347 (28.3%) | 28 (24.8%) | 217 (31.9%) | 194 (29.8%) |  |
| Missing | 13 (0.5%) | 3 (0.2%) | 1 (0.6%) | 5 (0.8%) | 4 (0.8%) |  |
| **quartile**  **UIC(ug/L)** | 154.40 [85.54, 277.20] | 144.50 [81.86, 261.09] | 134.47 [62.62, 186.68] | 198.94 [107.90, 306.82] | 150.39 [84.71, 281.59] | 0.47 |
| **quartile**  **Thyroid peroxidase antibodies (IU/mL)** | 0.60 [0.30, 1.60] | 0.70 [0.40, 1.50] | 0.50 [0.30, 0.90] | 0.60 [0.30, 1.70] | 0.70 [0.30, 1.89] | 0.02 |
| **quartile**  **TSH (mIU/L)** | 1.67 [1.12, 2.47] | 1.72 [1.13, 2.51] | 1.36 [0.97, 2.19] | 1.71 [1.15, 2.50] | 1.58 [1.05, 2.43] | 0.88 |
| **quartile**  **Thyroglobulin antibodies (IU/mL)** | 0.60 [0.60, 0.60] | 0.60 [0.60, 0.60] | 0.60 [0.60, 0.60] | 0.60 [0.60, 0.60] | 0.60 [0.60, 0.60] | 0.96 |
| **quartile**  **Thyroglobulin (ug/L)** | 9.74 [5.43, 16.46] | 9.74 [5.61, 16.81] | 11.65 [6.80, 17.46] | 9.29 [4.75, 15.88] | 9.85 [5.22, 16.02] | 0.31 |
| ^1^For continuous variables: survey-weighted Mean (SD) or survey-weighted Median(IQR), P-value was by survey-weighted linear regression (svyglm). For categorical variables: N-observe (survey-weighted percentage), P-value was by survey-weighted Chi-square test (svytable). | | | | | | |

Tables 1:

Baseline characteristics of participants in 2007–2012 (weighted). BMI: body mass index; UIC: urinary iodine concentration, a measure of iodine status.

Vitamin B6 supplementation indicates the use of vitamin B6 supplements by the participants over a 1-month period. Continuous variables are presented as weighted mean (SD), and categorical variables as weighted percentage.

|  |  | Model 1 | P Value | Model 2 | P Value | Model 3 | P Value |
| --- | --- | --- | --- | --- | --- | --- | --- |
| TGAb | Continuous | 0.12(-0.06, 0.30) | 0.2 | 0.10(-0.07, 0.28) | 0.2 | 0.10(-0.08, 0.27) | 0.3 |
|  | Q1 | Ref |  | Ref |  | Ref |  |
|  | Q2 | -5.8(-10, -1.5) | 0.009 | -4.5(-9.8, 0.87) | 0.1 | -3.3(-8.9, 2.3) | 0.2 |
|  | Q3 | 1.6(-6.5, 9.6) | 0.7 | -1.1(-8.4, 6.2) | 0.8 | -1.5(-8.6, 5.5) | 0.7 |
|  | Q4 | -0.55(-7.7, 6.6) | 0.9 | -1.5(-8.7, 5.7) | 0.7 | -2.3 (-9.1, 4.5) | 0.5 |
|  |  |  |  |  |  |  |  |
| T3 | Continuous | 0.00(0.00, 0.00) | 0.063 | 0.00(0.00, 0.00) | 0.2 | 0.00(0.00, 0.00) | 0.14 |
|  | Q1 | Ref |  | Ref |  | Ref |  |
|  | Q2 | -0.08(-0.17, 0.00) | 0.052 | -0.10(-0.18, -0.02) | 0.012 | -0.10(-0.18, -0.02) | 0.017 |
|  | Q3 | -0.08(-0.14, -0.03) | 0.005 | -0.03(-0.08, 0.02) | 0.2 | -0.03(-0.08, 0.01) | 0.2 |
|  | Q4 | -0.07(-0.13, -0.01) | 0.034 | -0.04(-0.10, 0.01) | 0.13 | -0.04(-0.10, 0.01) | 0.12 |
|  |  |  |  |  |  |  |  |
| T4 | Continuous | 0.00(-0.01, 0.00) | >0.9 | 0.00(-0.01, 0.00) | 0.7 | 0.00(-0.01, 0.00) | 0.8 |
|  | Q1 | Ref |  | Ref |  | Ref |  |
|  | Q2 | -0.43(-0.85, -0.01) | 0.045 | -0.17(-0.47, 0.12) | 0.12 | -0.15(-0.46, 0.16) | 0.2 |
|  | Q3 | 0.29(0.03, 0.56) | 0.032 | 0.10(-0.17, 0.37) | 0.5 | 0.09(-0.20, 0.39) | 0.5 |
|  | Q4 | -0.12(-0.41, 0.17) | 0.4 | -0.17(-0.47, 0.12) | 0.2 | -0.15(-0.46, 0.16) | 0.3 |
|  |  |  |  |  |  |  |  |
| TG | Continuous | 0.00(-0.03, 0.03) | >0.9 | -0.01(-0.05, 0.03) | 0.5 | -0.02(-0.08, 0.03) | 0.4 |
|  | Q1 | Ref |  | Ref |  | Ref |  |
|  | Q2 | 1.1(-3.4, 5.5) | 0.6 | 0.06(-5.0, 5.1) | >0.9 | 0.09(-5.5, 5.7) | >0.9 |
|  | Q3 | -1.2(-3.0, 0.74) | 0.2 | -2.1(-4.3, 0.06) | 0.056 | -2.1(-4.6, 0.35) | 0.088 |
|  | Q4 | 7.0(-5.8, 20) | 0.3 | 6.4(-5.7, 18) | 0.3 | 6.0(-4.9, 17) | 0.3 |
|  |  |  |  |  |  |  |  |
| TSH | Continuous | 0.00(0.00, 0.00) | 0.2 | 0.00(0.00, 0.00) | 0.092 | 0.00(0.00, 0.00) | 0.051 |
|  | Q1 | Ref |  | Ref |  | Ref |  |
|  | Q2 | 0.02(-0.47, 0.51) | >0.9 | 0.10(-0.39, 0.59) | 0.7 | 0.12(-0.38, 0.63) | 0.6 |
|  | Q3 | 0.16(-0.09, 0.41) | 0.2 | 0.06(-0.24, 0.35) | 0.7 | 0.04(-0.25, 0.34) | 0.8 |
|  | Q4 | -0.08(-0.25, 0.10) | 0.4 | -0.11(-0.28, 0.07) | 0.2 | -0.12(-0.29, 0.05) | 0.15 |
|  |  |  |  |  |  |  |  |
| TPOAb | Continuous | 0.01(-0.14, 0.16) | >0.9 | -0.01(-0.17, 0.15) | 0.9 | -0.02(-0.17, 0.13) | 0.8 |
|  | Q1 | Ref |  | Ref |  | Ref |  |
|  | Q2 | 1.8(-16, 19) | 0.8 | -2.7(-20, 15) | 0.8 | -1.6(-19, 16) | 0.9 |
|  | Q3 | 15(1.6, 29) | 0.029 | 18(2.0, 35) | 0.029 | 18(1.1, 34) | 0.038 |
|  | Q4 | 12(-2.7, 26) | 0.11 | 10(-5.0, 25) | 0.2 | 9.4(-5.8, 25) | 0.2 |
|  |  |  |  |  |  |  |  |
| TT3 | Continuous | 0.00(0.00, 0.00) | 0.2 | 0.00(0.00, 0.00) | 0.5 | 0.00(0.00, 0.00) | 0.4 |
|  | Q1 | Ref |  | Ref |  | Ref |  |
|  | Q2 | 0.03(-0.07, 0.14) | 0.5 | -0.02(-0.13, 0.09) | 0.7 | -0.02(-0.13, 0.09) | 0.7 |
|  | Q3 | -0.09(-0.14, -0.03) | 0.003 | -0.01(-0.06, 0.04) | 0.6 | -0.01(-0.06, 0.05) | 0.7 |
|  | Q4 | 0.02(-0.05, 0.09) | 0.6 | 0.04(-0.03, 0.10) | 0.3 | 0.04(-0.03, 0.11) | 0.2 |
|  |  |  |  |  |  |  |  |
| TT4 | Continuous | 0.00(0.00,0.00) | 0.5 | 0.00(0.00,0.00) | 0.8 | 0.00(0.00,0.00) | 0.8 |
|  | Q1 | Ref |  | Ref |  | Ref |  |
|  | Q2 | -0.04(-0.44,0.35) | 0.8 | -0.15(-0.57,0.28) | 0.5 | -0,15(-0.57,0.26)0.4 | 0.4 |
|  | Q3 | -0.01(-0.17,0.15) | >0.9 | -0.01(-0.18,0.16) | 0.9 | -0.03(-0.20,0.14) | 0.7 |
|  | Q4 | 0.17(-0.07,0.42) | 0.2 | 0.14(-0.11,0.38) | 0.3 | 0.13(-0.10,0.36) | 0.3 |

Tables 2

Model 1: unadjusted.

Model 2: adjusted for age, sex, ethnicity, education level, and UIC.

Model 3: adjusted for age, sex,ethnicity,education level,marital status, annual household income and poverty ratio, UIC, smoking, energy intake, dietary fibre, and BMI.
